# The Effectiveness of Overground Robot Exoskeleton Gait Training on Gait Outcomes, Balance, and Motor Function in Patients with Stroke: A Systematic Review and Meta-Analysis of Randomized Controlled Trials

**DOI:** 10.3390/brainsci14080834

**Published:** 2024-08-19

**Authors:** Myoung-Ho Lee, Ming-Yu Tian, Myoung-Kwon Kim

**Affiliations:** 1 Department of Rehabilitation Sciences, Graduate School, Daegu University, Jillyang, Gyeongsan 712-714, Gyeongbuk, Republic of Korea; hotayaaa@gmail.com (M.-H.L.); mingyutian5@gmail.com (M.-Y.T.); 2 Department of Physical Therapy, College of Rehabilitation Sciences, Daegu University, Jillyang, Gyeongsan 712-714, Gyeongbuk, Republic of Korea

**Keywords:** balance, gait training, overground, robot exoskeleton, stroke

## Abstract

Objective: This study aimed to investigate the effects of overground robot exoskeleton gait training on gait outcomes, balance, and motor function in patients with stroke. Methods: Following the PRISMA guidelines, literature searches were performed in the PubMed, EMBASE, Cochrane Central Register of Controlled Trials, SCOPUS, Ovid-LWW, and RISS databases. A total of 504 articles were identified, of which 19 were included for analysis after application of the inclusion and exclusion criteria. The included literature was qualitatively evaluated using the PEDro scale, while the Egger’s regression, funnel plot, and trim-and-fill methods were applied to assess and adjust for publication bias. Results: The averaged PEDro score was 6.21 points, indicating a high level of methodological quality. In the analysis based on dependent variables, higher effect sizes were observed in the following ascending order: gait speed (g = 0.26), motor function (g = 0.21), gait ability (g = 0.18), Timed Up and Go Test (g = −0.15), gait endurance (g = 0.11), and Berg Balance Scale (g = 0.05). Subgroup analyses further revealed significant differences in Asian populations (g = 0.26), sessions lasting longer than 30 min (g = 0.37), training frequency of three times per week or less (g = 0.38), and training duration of four weeks or less (g = 0.25). Overall, the results of this study indicate that overground robot exoskeleton gait training is effective at improving gait speed in patients with stroke, particularly when the sessions exceed 30 min, are conducted three times or less per week, and last for four weeks or less. Conclusion: our results suggest that training is an effective intervention for patients with stroke, provided that appropriate goal-setting and intensity and overground robot exoskeleton gait are applied.

## 1. Introduction

In recent years, ground gait systems for patients with stroke have been developed [1], while robot-assisted therapy has emerged as a promising technology in recent decades. In this context, robotic devices are used as therapeutic approaches to treat neurological injuries and support stroke rehabilitation training [2]. With recent advances in technology, robot-assisted training is increasingly being used in poststroke rehabilitation of balance, gait, and daily function [3].

Devices for motor function recovery can be divided into the following two types: end-effector robots and exoskeleton robots [4]. Robot-assisted gait strategies can further be classified into static methods, which restrict users to walking in a fixed environment, and ground-based methods, which allow users to move freely within the environment [5]. Exoskeleton robots comprise an external mechatronic system with joints matching those of the human skeleton, allowing the exoskeleton to move in parallel with the skeleton of patients with stroke [6]. While the construction of exoskeleton robots is complex, they can allow for the direct control of individual joints, reducing adverse effects and abnormal posture. Because of the safety and high efficiency of robotic equipment, the adaptation to treatment in patients with stroke is favorable [7].

Overground robot-assisted gait training (RAGT) is a robotic device that differs from treadmill-based RAGT, as it allows patients to explore their environment while walking on solid ground using wearable exoskeletons, providing almost normal proprioception [8]. This approach enables patients to actively participate in tasks such as balance control, weight shifting, and muscle activation, which are crucial for safe gait reconstruction and recovery [9,10]. Additionally, it controls and measures exercise intensity, measuring kinematic and kinetic data to allow sensitive and reliable evaluation of gait form [11]. These techniques can enable patients with stroke to quickly recover gait function, resume daily activities sooner, and regain gait independence [12]. Moreover, this rehabilitation has been found to be effective for patients who cannot maintain an upright posture, as it provides overground gait training compared to traditional treadmill training, enabling posture and balance exercises [13].

We identified systematic review and meta-analysis articles assessing robot-assisted gait training and balance and gait ability [14,15,16,17,18,19,20,21,22]. Among these, Bruni et al. [15] reported that combining robot-assisted gait training with conventional physical therapy offers significant benefits, while Moucheboeuf et al. [20] further showed that robot-assisted gait training combined with body weight support training and general physical therapy are effective interventions for gait recovery after stroke. Conversely, Postol et al. [21] reported no differences in the 6 min walk test and the 10 m walk test between robot therapy and control groups. Similarly, Tedla et al. [22] reported no significant differences between robot-assisted gait training and conventional gait training. A meta-analysis by Wang et al. [23] found that applying more than 10 h of robot-assisted therapy to patients with stroke significantly improved balance; however, some studies reported that the effect on balance was not significant [14,17,21]. Among these reviews, intervention trainings, involving various electronic devices as mixed interventions [19,20,22] and static interventions [14,15,17,23], were assessed not only in patients with stroke but also in patients with brain injuries involving neurological disorders [21]. Further, highly heterogeneous reviews were included [16]. Given this wide disparity, the effects of overground robot gait training on gait-related outcomes and balance are uncertain. The use of robot-assisted gait rehabilitation using treadmill-based robots for motor control in stroke rehabilitation has increased; however, treadmill-based robot-assisted gait training conditions differ from actual overground gait training conditions. As such, the improvement in gait ability following treadmill-based robot training may not be directly interpreted as enhanced overground gait [24].

Research on overground robot-assisted gait training for patients with stroke is extremely limited due to its high complexity. Despite the increasing number of randomized controlled trials, systematic reviews and meta-analyses of the effects of standalone overground robotic exoskeleton or standalone overground robot-assisted gait training combined with traditional rehabilitation have not yet been conducted. Additionally, no review has yet focused on motor function. Therefore, the aim of this paper was to evaluate the effects of overground robot exoskeleton gait training on gait outcomes, balance, and motor function in patients with stroke, as well as to provide evidence for its potential as an effective rehabilitation method. To achieve this, we conducted a systematic review and meta-analysis to comprehensively assess the results of various clinical studies, aiming to identify the specific benefits and impacts of overground robot-assisted gait training in stroke rehabilitation.

## 2. Methods

### 2.1. Research Design

This study analyzed the effects of robot-assisted gait training on patients with stroke using a meta-analysis. The study procedures were conducted in accordance with the Preferred Reporting Items for Systematic Review and Meta-Analyses (PRISMA) Flowchart [25]. The study was designed by researchers with sufficient knowledge of systematic review and meta-analysis, and it was carried out in collaboration with two physical therapists holding doctoral degrees in physical therapy.

### 2.2. Information Source

The literature search was carried out from January 2024 to February 2024 in the following databases: PubMed, EMBASE, Cochrane Central Register of Controlled Trials, Scopus, and Ovid-LWW. Additionally, documents published on the RISS (Research Information Sharing Service), a repository for gray literature including master’s and doctoral theses, were also searched. Furthermore, relevant articles were manually searched in specific journals, as well as in the reference lists of retrieved studies and systematic reviews.

### 2.3. Search Strategy

A 3-step search strategy was employed to ensure the comprehensiveness of the search. The first phase involved a preliminary search in PubMed to identify existing reviews, suitable keywords, and index terms. Related terms and synonyms were subsequently identified based on the following 3 key concepts: “Stroke”, “Exoskeleton device”, and “Overground gait training”. In the second phase, ongoing trials were searched in 4 clinical trial databases, which included the EMBASE, Cochrane Central Register of Controlled Trials, Scopus, and Ovid-LWW. Finally, the third phase involved identifying studies using other methods, such as gray literature, specific journals, and hand-searches of reference lists.

### 2.4. Criteria for Inclusion and Exclusion of Data

#### 2.4.1. Inclusion Criteria for the Current Study

(1) Randomized controlled trials (RCTs).
(2) Studies assessing participants who met the clinical diagnosis criteria for stroke or were diagnosed with stroke by MRI or CT, without comorbidities such as severe cognitive impairment, heart failure, or exercise contraindications.
(3) No restrictions were placed on country, age, gender, or treatment duration.
(4) Intervention using robot-assisted gait training, either alone or combined with other treatments, while control groups underwent conventional gait training, including physical therapy or other common rehabilitation approaches.
(5) (Outcome measures included assessments of gait outcomes, balance, or motor function obtained through any measurement scale.
(6) Studies published exclusively in English.

#### 2.4.2. Exclusion Criteria for the Current Study

(1) Single-group experimental designs without a control group.
(2) Nonexperimental studies, such as observational, case studies, qualitative research, animal experiments, and duplicate publications reporting the same results.
(3) Gray literature papers (abstracts and posters) without peer review.
(4) Studies lacking sufficient data for effect size analysis.
(5) Reported results containing errors or inaccuracies in tables or figures.

### 2.5. Selection Process

The study selection process was conducted based on the PRISMA 4-phase flow diagram. Firstly, duplicates were identified and removed using EndNote 20 software. Additionally, any duplicates not identified by the software were manually removed. Next, two reviewers (M.-H.L. and M.-Y.T.) independently screened the titles and abstracts of the studies retrieved from the search, removing irrelevant studies based on predefined eligibility criteria. Subsequently, two independent reviewers examined the full-text articles of the selected studies to ascertain their inclusion status. All disagreements between the two reviewers were resolved through discussion or with the assistance of a third reviewer (M.-K.K.). The Cohen’s kappa coefficient (k) between the two reviewers was 0.80, indicating a substantial level of inter-rater reliability.

### 2.6. Data Collection Process and Data Items

The form for data extraction was created based on the *Cochrane Handbook for Systematic Reviews of Interventions* [26]. Two independent reviewers (M.-H.L. and M.-Y.T.) conducted a pilot test on five randomly chosen studies to evaluate the accuracy in the extraction of all pertinent information [27]. Two reviewers compared and discussed the pilot test results, achieving an almost perfect agreement with a Cohen’s kappa coefficient of 0.85. Throughout the review process, extracted data were recorded and managed using Microsoft Excel 365. The data items included authors, publication year, study design, type of stroke, sample size, gender, average age, stroke phase, training type, control group, walking-related outcomes, measurement type, units, intention-to-treat analysis, management of missing data, and attrition rates. For training descriptions, the items covered baseline walking ability, type and weight of the robotic device, training environment, cointerventions, control group settings, training intensity and duration, follow-up, and assessment. The respective study authors were contacted via email to verify missing data and to request additional information.

### 2.7. Data Analysis

The data analysis for this study was conducted using the Comprehensive Meta-Analysis software (CMA V4). For effect size analysis, the number of subjects, means, and standard deviations for both the experimental and control groups were extracted from each of the included studies. When the results were reported as the median and interquartile range or minimal–maximal range, they were converted to the mean and standard deviation for analysis. Additionally, studies by Yeung [28] and Jayaraman [29], which reported outcomes as changes, utilized change scores and standard deviations to calculate effect sizes.

### 2.8. Risk of Bias Assessment

The PEDro scale (Physiotherapy Evidence Database scale) was utilized to assess the quality of the selected studies. The PEDro scale comprises 11 items, with scores determined by whether the included studies meet these items. Each satisfied item (excluding the first one) contributes 1 point to the total score, which ranges from 0 to 10 points. The overall score is categorized into the following three levels: (1) high quality (score of 6–10), (2) fair quality (score of 4–5), and (3) poor quality (score ≤ 3). Compared to the traditional Jadad scale, the PEDro scale offers higher levels of reliability and validity [30]. The details of the contents for the PEDro scale are shown in Table 1.

### 2.9. Reporting Bias Assessment

Ten or more studies were pooled for an outcome; thus, visualization of funnel plots was employed to assess small-study effects and publication bias [31]. Egger’s regression test was performed using CMA software to test for publication bias, with *p* < 0.05 indicating statistically significant publication bias [32].

### 2.10. Synthesis Methods

A meta-analysis was conducted using Review Manager v5.4 to integrate the results. The findings were visually interpreted for heterogeneity and significance using a forest plot. The inverse-variance method was applied to calculate the standardized mean difference (SMD) for continuous outcomes [33]. The overall effect of overground robot exoskeleton gait training was analyzed using Z-statistics at a significance level of *p* < 0.05, and the effect size of the results was based on the standardized mean difference (SMD) or Cohen’s d. Hedge’s g, a corrected effect size based on Cohen’s d, was also calculated [34]. As Cohen’s d values can overestimate effect sizes when the sample size is small, correction is necessary [35]. According to Bernard et al. [36], it is recommended to convert Cohen’s d values to Hedge’s g values when studies with large and small samples are mixed. Therefore, in this study, Hedge’s g values were calculated to summarize the effect size, with the resulting effect sizes categorized as follows: small (<0.3), moderate (0.3–0.8), large (0.8–1.3), and very large (>1.3) [37].

The heterogeneity of the results was evaluated based on the I^2^ statistic and chi-square tests (χ2). The degree of heterogeneity was classified as low (≤40%), moderate (30−60%), substantial (50−90%), or very high (75−100%) [33]. An χ2 value with *p* < 0.10 indicated statistical significance of heterogeneity.

Subgroup analyses were conducted to explore the heterogeneity and compare the effects of subsets in identifying the preferred training design for overground robot exoskeleton training [33]. Predefined subgroups included the region and phase of stroke, length of training session, and frequency and duration of training. A random-effects univariate meta-regression model was performed using CMA software to examine the effect of covariates on the training effect.

## 3. Results

### 3.1. Study Selection

**Figure 1:**
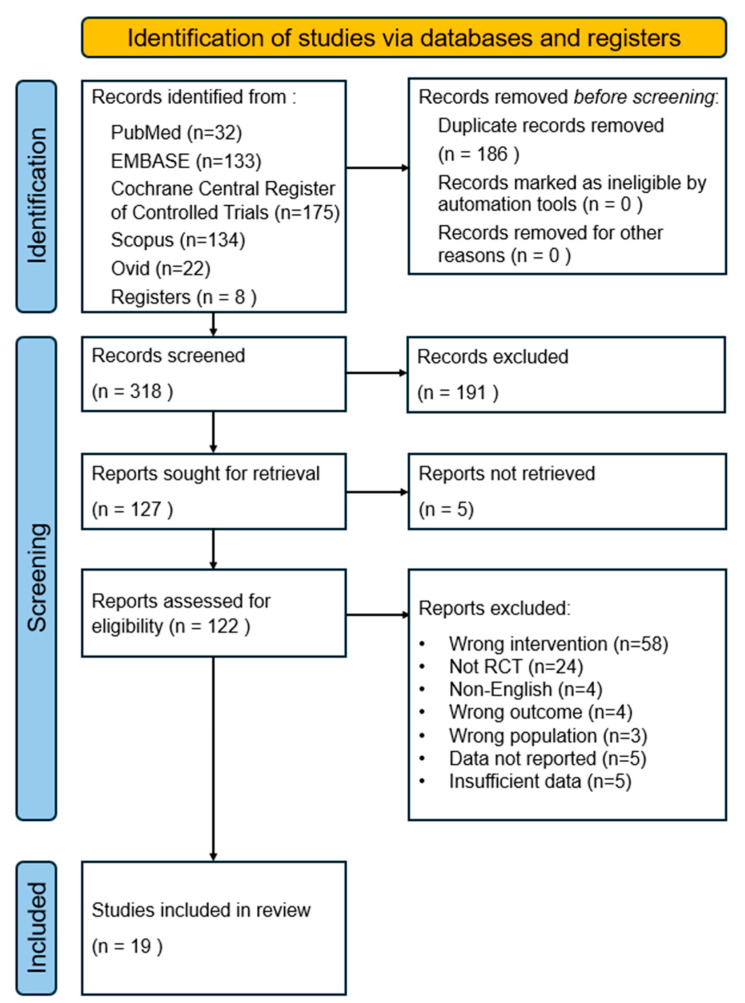
PRISMA flow chart diagram of study selection.

The study selection process is summarized in the PRISMA flowchart. A total of 496 records were identified from electronic searches of five databases. Additional articles were identified through other clinical trials, gray literature, reference lists, and hand-searched journals (n = 8). Duplicate records were manually removed using EndNote software, resulting in the removal of 186 duplicate articles. Subsequently, 318 records were screened by the title and abstract based on the eligibility criteria described earlier, resulting in the exclusion of 191 records. Five studies were not retrieved, and a comprehensive review of the full text of 122 studies excluded 103 of them. The reasons for exclusion were as follows: incorrect intervention (n = 58), non-RCT (n = 24), non-English language (n = 4), wrong outcome (n = 4), wrong population (n = 3), data not reported (n = 5), and insufficient data (n = 5). Finally, 19 articles were selected for inclusion in the meta-analysis. Figure 1 depicts the flowchart.

### 3.2. Characteristics of the Included Studies

Table 2 summarizes the characteristics of the included 19 RCTs, targeting a total of 756 patients with stroke from eight countries, including Japan (n = 6), South Korea (n = 4), the United States (n = 2), Italy (n = 2), China (n = 2), Canada (n = 1), the United Kingdom (n = 1), and Hong Kong (n = 1). All RCTs were two-arm trials, and five were pilot studies. The studies recruited participants in the acute (n = 2), subacute (n = 9), and chronic (n = 8) stroke stages. The sample sizes of the individual studies varied from 22 to 130 participants. The mean age of the participants ranged from 48.33 to 76.8 years old. The data are shown in Table 2.

### 3.3. Description of the Overground Robotic Exoskeleton Training

Overground RE training was conducted using various types of overground RE training, as follows: Hybrid Assistive Limb training (n = 5), EksoGT powered exoskeleton training (n = 2), Stride Management Assistant system training (n = 2), Alter G Bionic Leg training (n = 2), BEAR-H1 assisted gait training (n = 2), Exowalk (n = 2), Ekso™ (n = 1), Gait Enhancing and Motivating System (n = 1), Dynamixel MX (n = 1), and SUBAR exoskeleton (n = 1).

The duration per session ranged from 20 to 60 min. The intervention period ranged from 10 days to 10 weeks, with a frequency of 2 to 5 sessions per week. Both the overground RE training group and the control group had similar session lengths, frequencies, and durations. In seven studies, follow-up assessments were specifically conducted at 2 weeks (n = 1), 22 weeks (n = 1), 2 months (n = 1), 3 months (n = 2), 1 month and 3 months (n = 1), and 6 months (n = 1).

### 3.4. Risk of Bias

The evaluation of the quality of studies using PEDro scores yielded the following results: a total of 19 studies were included, with an average PEDro score of 6.21 points. Subject and therapist blinding was associated with an overall high risk of bias, as blinding patients to the physical therapy intervention was not feasible (100%). In more than half of the studies, the allocation concealment was unclear, as they either did not provide sufficient information or adequately address the outcome. Five articles reported attrition rates of over 15%, while eight articles used intention-to-treat analysis. None of the articles were rated as poor quality (score ≤ 3); six articles were rated as fair quality (score of 4–5), and thirteen articles were rated as high quality (score of 6–10). This study’s researchers carefully reviewed and determined these outcomes under the supervision of two PhDs in physical therapy, including the lead supervisor. The data are shown in Table 3.

### 3.5. Effect Size Analysis Results

#### 3.5.1. Gait Speed

**Figure 2:**
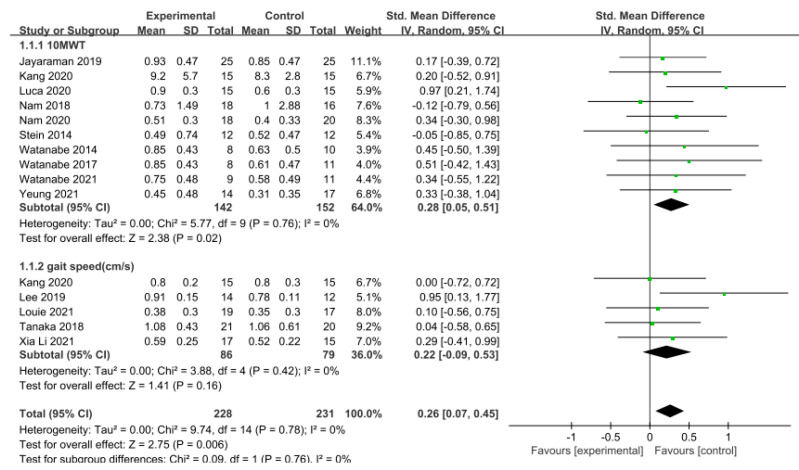
Forest plot of gait speed [28,29,39,40,42,43,45,46,47,48,49,50,51,52].

**Figure 3:**
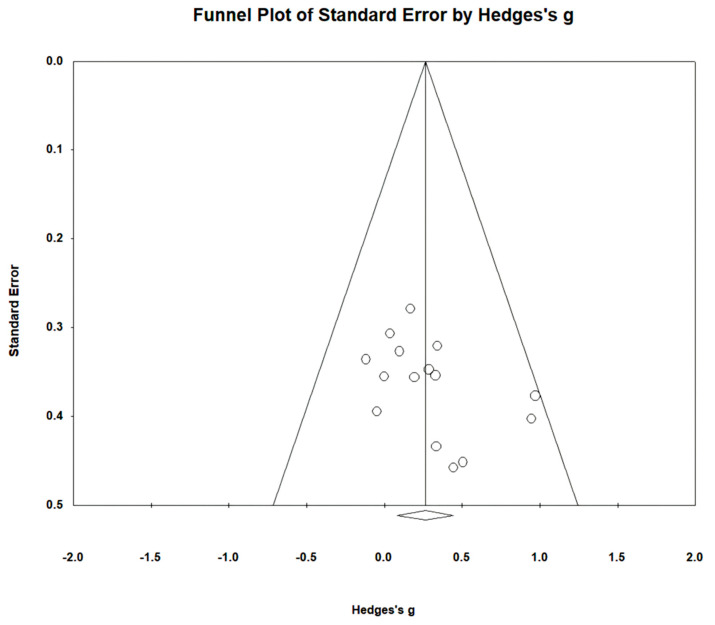
Funnel plot of gait speed.

Gait speed was measured using the 10 m walking test (10MWT) and gait analysis in 15 studies involving 459 participants. The homogeneity test indicated low levels of heterogeneity (Chi^2^ = 9.74, *p*-value = 0.78, I^2^ = 0%), and a random-effects model was therefore employed. In the meta-analysis, a small effect size of 0.26 (95% CI = 0.07, 0.45) was observed, with statistical significance (*p* < 0.05). Egger’s regression test was employed to statistically analyze publication bias, revealing no evidence of publication bias (*p*-value = 0.09, 95% CI = −0.56, 5.99). The data are shown in Table 4 and depicted in Figure 2 and Figure 3.

A series of subgroup analyses was conducted on various factors, including region, phase of stroke, length of training session, and frequency and duration of training, focusing on their impact on gait speed. In studies conducted within Asian regions, the effect size was 0.26, which was slightly lower compared to those conducted in non-Asian regions (effect size = 0.28), but still demonstrated a significant difference (*p* < 0.05). Notably, there were no studies on acute stroke. Conversely, chronic phases (g = 0.28) exhibited a larger effect size compared to subacute phases (g = 0.25). Moreover, when the training session lasted less than or equal to 30 min (g = 0.18), a smaller effect size was observed in contrast to sessions lasting more than 30 min (g = 0.37), indicating a significant difference. Regarding the training frequency, studies conducted with a frequency of three sessions per week or less (g = 0.38) displayed a larger effect size than those conducted with a frequency exceeding three sessions per week (g = 0.12), revealing a significant difference. Furthermore, studies with a training duration of four weeks or less (g = 0.25) exhibited a lower effect size compared to those longer more than four weeks (g = 0.28), although a significant difference was evident. The data are shown in Table 5. 

#### 3.5.2. Gait Endurance

**Figure 4:**
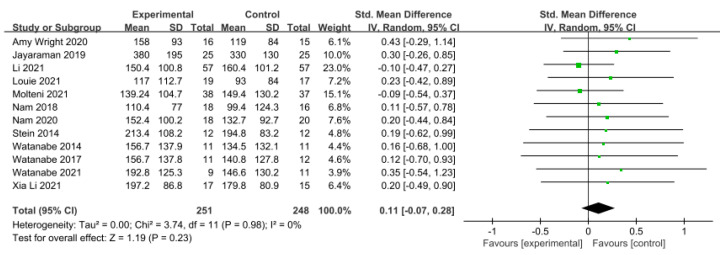
Forest plot of gait endurance [29,38,41,42,44,45,46,47,49,50,51,52].

**Figure 5:**
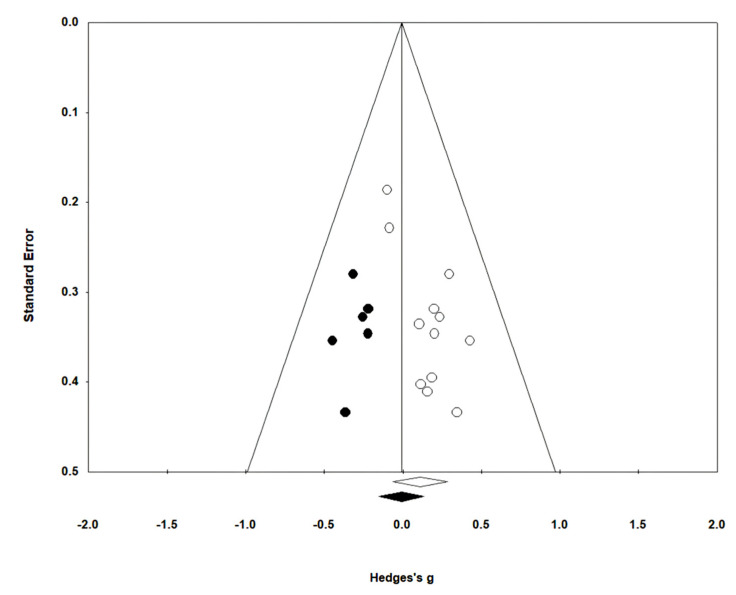
Funnel plot of gait endurance.

Gait endurance was measured using the 6 min walking test (6MWT) in 12 studies involving 499 participants. The homogeneity test indicated low levels of heterogeneity (Chi^2^ = 3.74, *p*-value = 0.98, I^2^ = 0%), and a random-effects model was employed. In the meta-analysis, a very small effect size of 0.11 (95% CI = −0.07, 0.28) was observed, with no statistical significance. Egger’s regression test was employed to statistically analyze publication bias, revealing the presence of publication bias (*p*-value = 0.003, 95% CI = 0.68, 2.63). Subsequently, Duval and Tweedie’s trim-and-fill method was applied, resulting in the addition of 6 studies. This led to a shift in the effect size from 0.11 to −0.009 (CI = −0.15, 0.13). The data are shown in Table 6 and depicted in Figure 4 and Figure 5.

#### 3.5.3. Gait Ability

**Figure 6:**
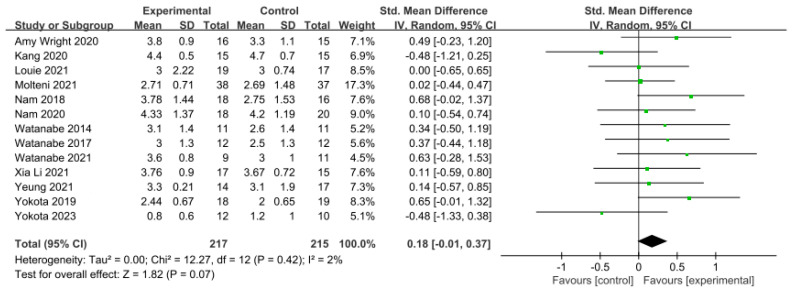
Forest plot of gait ability [28,38,39,42,44,45,46,49,50,51,52,53,54].

**Figure 7:**
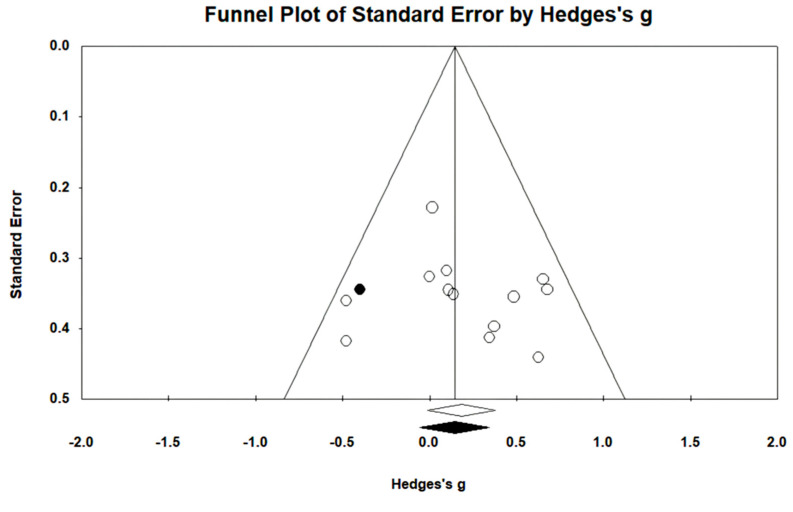
Funnel plot of gait ability.

In 13 studies involving 432 participants, gait ability was measured using the functional ambulation category (FAC). The homogeneity test indicated low levels of heterogeneity (Chi^2^ = 12.27, *p*-value = 0.42, I^2^ = 2%), and a random-effects model was employed. In the meta-analysis, a small effect size of 0.18 (95% CI = −0.01, 0.37) was observed, with no statistical significance. Egger’s regression test was employed to statistically analyze publication bias, revealing no publication bias (*p*-value = 0.588, 95% CI = −2.82, 4.73). For the sensitivity analysis, Duval and Tweedie’s trim-and-fill method was used, resulting in the addition of one study. This led to a slight change in the effect size from 0.18 to 0.14 (CI = −0.05, 0.34). The data are shown in Table 7 and depicted in Figure 6 and Figure 7.

#### 3.5.4. Balance

Balance was assessed using the Berg Balance Scale (BBS) in eight studies involving 274 participants, while seven studies including 172 participants utilized the Timed Up and Go Test (TUG).

In the studies using the BBS, a low level of heterogeneity was found based on the homogeneity test (Chi^2^ = 9.09, *p*-value = 0.25, I^2^ = 23%), and a random-effects model was therefore used. The effect size was 0.05 (95% CI = −0.23, 0.32), indicating a very small effect size with no statistical significance. Because of the limited number of included studies, statistical tests for publication bias and visual analysis using funnel plots were not conducted.

**Figure 8:**
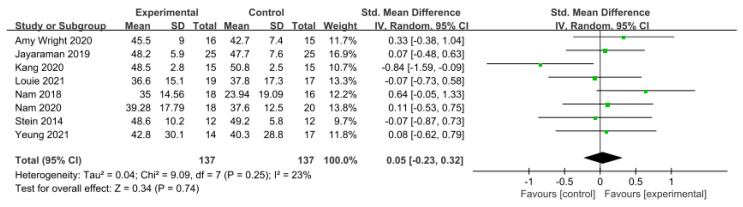
Forest plot of balance−Berg Balance Scale [28,29,38,39,42,45,46,47].

**Figure 9:**
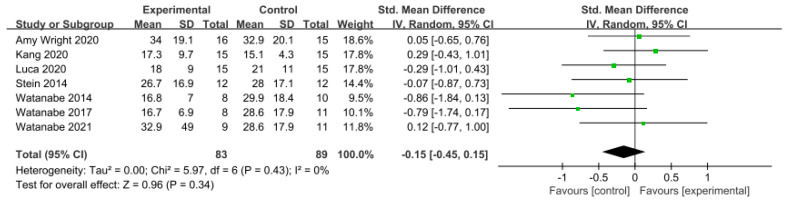
Forest plot of balance−Timed Up and Go Test [38,39,43,47,49,50,51].

Similarly, in the studies utilizing the TUG Test, a low level of heterogeneity was observed based on the homogeneity test (Chi^2^ = 5.97, *p*-value = 0.43, I^2^ = 0%), and a random-effects model was therefore employed. The effect size was −0.15 (95% CI = −0.45, 0.15), indicating a very small effect size, with no statistical significance. Again, because of the limited number of included studies, statistical tests for publication bias and visual analysis using funnel plots were not conducted. The data are shown in Table 8 and depicted in Figure 8 and Figure 9.

#### 3.5.5. Motor Function

**Figure 10:**
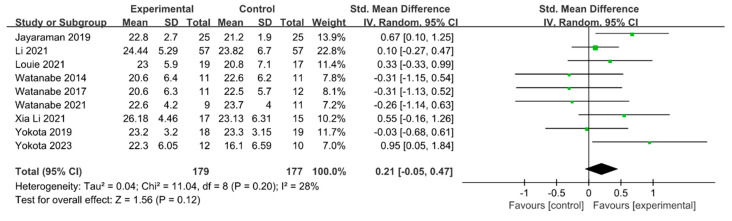
Forest plot of motor function [29,41,42,49,50,51,52,53,54].

Motor function was measured using the Fugl–Meyer Assessment—Lower Extremity (FMA-LE) in nine studies involving 356 participants. The homogeneity test indicated low levels of heterogeneity (Chi^2^ = 11.04, *p*-value = 0.20, I^2^ = 28%), and a random-effects model was employed. In the meta-analysis, a small effect size of 0.21 (95% CI = −0.05, 0.47) was observed, with no statistical significance. Because of the limited number of included studies, statistical tests for publication bias and visual analysis using funnel plots were not conducted. The data are shown in Table 9 and depicted in Figure 10.

## 4. Discussion

To our knowledge, this study is the first review to comprehensively evaluate and demonstrate the outcomes of gait, balance, and motor function achievable through gait training with an overground robot exoskeleton. This review analyzed 19 randomized controlled trials conducted in eight countries involving a total of 756 patients with stroke. The results of this meta-analysis overall showed a significant improvement in gait speed with overground robot exoskeleton gait training, with a small effect size. However, no significant differences were observed in gait endurance, gait ability, balance, or motor function.

### 4.1. Classification According to Dependent Variables

The analysis of the effect sizes based on each dependent variable revealed small effect sizes for gait speed and motor function and very small effect sizes for other variables. Examining the findings of this study, it can be concluded that overground robot exoskeleton gait training is relatively more effective for gait speed and motor function compared to other objectives in patients with stroke.

Approximately one-third of patients with stroke experience apathy [55], defined as reduced motivation [56]. In stroke rehabilitation, it is difficult to motivate patients to receive task-oriented training for functional recovery, particularly for patients with severe disabilities. Louie et al. [42] emphasized the importance of considering the emotional and psychological benefits of practicing standing and walking in patients post-stroke. Louie et al. [42] further stated that it is important to consider the emotional and psychological benefits of standing and walking practice in this patient population, while Jang [57] suggested that performing gait on the ground using a gait assistance robot with configurable training intensity is a promising strategy. It has further been reported that this intervention has a positive effect on improving the gait ability of patients with stroke, as it allows for the feeling of independent gait, rather than gait depending on the device. The observed changes in gait outcomes in this study suggest that patients with stroke may benefit both clinically and emotionally from gait training with appropriate motivation.

An advantage of rehabilitation robots for gait is their ability to reduce the involvement of therapists in training patients to perform repetitive movements, allowing for control over the quantity and intensity of therapy. Additionally, depending on the model, these robots can be designed to support body weight, thus enabling safe implementation of gait rehabilitation therapy [58]. However, there have been concerns raised regarding the lack of realism in gait rehabilitation robots. For example, these robots often only allow for the practice of preprogrammed gait patterns, which may pose challenges in providing realistic sensory-motor input. To address this issue, some researchers have suggested developing therapies that integrate virtual reality, aiming to provide more realistic and engaging rehabilitation experiences [59]. For example, Jang [57] reported that using a wearable robot with adjustable training intensity for gait assistance could positively impact the gait ability of patients with stroke. By allowing patients to adjust training intensity, the device provided a sensation of performing gait independently, rather than relying on the device, potentially enhancing the sense of independence during gait. Recent exoskeleton control systems have become capable of fine adjustments, allowing for the setting of various degrees of freedom exercises tailored to the assist-as-needed approach [60]. This enables intensive and repetitive training of lower limb movements closely mimicking natural human motion [61]. Because of the exoskeleton’s capabilities, patients with stroke have been shown to be able to adjust the illusionary muscle activation, aiding in the effective restoration of gait patterns [10]. This adjustment led to improvements in gait patterns, with adjustment of the symmetry ratio of gait, which is classified as a sensitive measure of recovery [62], making it an important factor in facilitating gait [63].

The dependent variable that yielded a significant difference in this study’s integrated analysis was gait speed, indicating a small effect size of 0.26 (95% CI: 0.08, 0.44). The reason for these results can be understood through prior research confirming the effectiveness of overground robot exoskeleton gait training in improving gait speed [64,65]. Indeed, training may be attributed to the exoskeleton’s built-in multisensory system, which promotes interaction between humans and robots through kinematic and kinetic feedback to induce the accurate relearning of motor processes [66]. Such training can further enable individuals to achieve gait patterns that enhance their gait speed and ability [60].

In the subgroup analysis of gait speed, variables such as region, phase of stroke, length of training session, frequency of training, and duration of training were analyzed. Overall, we identified 11 relevant studies conducted in Asian regions and 4 in non-Asian regions, indicating active research in Asia. The effect sizes were 0.26 (95% CI: 0.03, 0.48) for Asian studies and 0.28 (95% CI: −0.13, 0.68) for non-Asian studies. Although the effect size appears higher for non-Asian regions, more studies were conducted in Asia and significant differences were found. There were no studies investigating the acute phase of stroke; however, seven studies focused on the subacute phase, while eight focused on the chronic phase. The effect sizes for each were 0.25 (95% CI: −0.04, 0.53) and 0.28 (95% CI: 0.01, 0.55), respectively, indicating small effect sizes. Regarding the length of training session, there were eight studies with sessions lasting 30 min or less and six studies with sessions lasting over 30 min. The effect sizes for each were 0.18 (95% CI: −0.07, 0.43) and 0.37 (95% CI: 0.05, 0.70), respectively. There was a significant difference in effect size, with sessions lasting over 30 min, showing a greater effect size. In the frequency of training, there were nine studies with training frequencies of three times a week or less and six studies with training frequencies exceeding three times a week. The effect sizes for each were 0.38 (95% CI: 0.12, 0.63) and 0.12 (95% CI: −0.15, 0.40), respectively. A significant difference was observed, with training frequencies of three times a week or less demonstrating a larger effect size. Regarding the duration of training, there were 10 studies with training periods of 4 weeks or less and 5 studies with training periods exceeding 4 weeks. The effect sizes for each were 0.25 (95% CI: 0.02, 0.48) and 0.28 (95% CI: −0.03, 0.60), respectively. Although the effect size for training periods of 4 weeks or less was smaller than that for training periods exceeding 4 weeks, a significant difference was still found because of the larger number of studies. Based on the results of this study, it can be concluded that when applying robot-assisted gait training, it is beneficial to extend the treatment duration and adjust the training frequency, focusing intensively on the initial phase of robot-assisted gait therapy.

Regarding gait speed, there is a need to expand research on acute patients with stroke. Additional research is further needed to examine differences in patient fatigue and adaptation to treatment based on training duration and frequency. Furthermore, since the effects of long-duration training are not yet clear, research to determine the impacts of long-duration overground robot exoskeleton gait training is also necessary.

For gait endurance, the effect size was 0.23 (95% CI: −0.07, 0.28), indicating a small effect size. However, publication bias was detected through Egger’s regression test. Using the trim-and-fill method to correct for this bias, the effect size was adjusted to −0.009 (95% CI: −0.15, 0.13), after adjusting six studies. The adjusted results of this study, accounting for publication bias, indicate that overground robot exoskeleton gait training has a minimal effect on gait endurance. This finding is consistent with the results of Mehrholz et al. [19].

Possible explanations for this could be that the patients with stroke participating in the experiment may have been in different phases of stroke, which could be associated with varying states of sensory-motor, visual, balance, pain, mood, and cognitive impairments [67]. According to past research, individual factors, such as age and prestroke activity, may further influence the walking endurance of patients with stroke [68]. However, such inferences are only possible in these cases. Therefore, further investigation is needed to explore the factors that influence gait endurance after overground robot exoskeleton gait training.

For gait ability, the effect size was 0.18 (95% CI: −0.01, 0.37), indicating a very small effect size, while no publication bias was detected. To conduct the sensitivity analysis, one study was adjusted using the trim-and-fill method, resulting in an adjusted effect size of 0.14 (95% CI: −0.05, 0.34). Nolan et al. [69] reported that patients with stroke receiving gait training with overground robot exoskeletons made only minimal progress during walking with assistive observation, while Mehrholz et al. [19] reported varying results in gait ability based on 23 studies, suggesting a higher likelihood of independent gait when using gait-assistive robots, although the outcomes differed across studies. In contrast, Bruni et al. [15] reported in their systematic review and meta-analysis that there was no evidence to suggest that robot exoskeletons were more effective than conventional therapy in patients with subacute stroke. Although our study also showed a very small effect size in gait ability, no significant difference was observed. Therefore, to clearly assess the training effects of ground-based gait training using exoskeleton robots, multicenter studies targeting a relatively larger number of participants are needed.

Two tools were used to measure balance. In the BBS, the effect size was 0.05 (95% CI: −0.23, 0.32), indicating a very small effect size. In the TUG Test, the effect size was −0.15 (95% CI: −0.45, 0.15). Most attempts to directly evaluate balance considered the BBS. However, this score primarily assesses balance in static environments or, in a few dynamic assessments, is based on single-task performance. Conversely, the TUG Test, depending on its execution, can demonstrate how balance is maintained in a more complex environment, with various anticipated disturbances being considered and countered [70]. Furthermore, while the BBS is highly reliable, it may not detect small changes in balance [71]. Therefore, consideration both the BBS and TUG in combination allows for the reliable assessment of both static and dynamic balance.

In half of the included studies, overground robot exoskeleton gait training was shown to be superior to conventional gait training in terms of BBS or TUG outcomes. However, previous studies have shown that both RAGT and traditional rehabilitation improve balance in patients with stroke without significant differences among groups [28,72,73,74,75,76]. One possible explanation for this is the significant impact of neuroplasticity in patients undergoing robot exoskeleton gait training. In the study by Kim et al. [77], an improvement in neuroplasticity was reported in patients with stroke undergoing end-effector RAGT, although no clear superiority of robot training over conventional therapy was found. The mechanisms regulating neuroplasticity are still not fully understood, and it therefore remains challenging to establish a correlation between robot rehabilitation and enhanced neuroplasticity. Additionally, there have been several studies emphasizing improvements in neuroplasticity using nonrobotic approaches, such as high-intensity interval training and neurorestorative techniques [78,79]. Considering these findings, a combined approach of robot exoskeleton gait training and conventional therapy may yield better results by enhancing neuroplasticity with both techniques. Overall, further research is needed to better understand the mechanisms and correlations between neuroplasticity and robot-assisted rehabilitation.

For the motor function domain, the effect size was 0.21 (95% CI: −0.05, 0.47), indicating a small effect size. The FMA scale is a tool developed to measure sensorimotor recovery in patients with stroke, based on a sequential concept of motor recovery stages in hemiplegic patients with stroke [80]. In one related study, Chang et al. [81] reported that robot-assisted training using an exoskeleton robot could increase the maximum oxygen consumption (VO2 max) of patients with stroke by up to 12.8%, thereby enhancing cardiopulmonary function and significantly improving lower limb muscle strength by promoting lower limb blood circulation. The improvement in gait and functional independence suggests the importance of spontaneous biological recovery processes poststroke, indicating not only simple recovery, but also the need to consider scales evaluating better daily activities or achieving independent gait.

Hemiplegic gait typically manifests as a reduced movement ability on the affected side during the swing phase, resulting in prolongation of the swing time on the affected side and support time on the unaffected side [82,83]. When balance control is weakened, both the support phase on the affected side and the swing phase on the unaffected side are significantly shortened [84]. In one study, Husemann et al. [85] reported that patients with stroke showed improved support and swing abilities on the affected side with enhanced strength, balance, and motor function through robot-assisted gait training. Overall, these findings suggest that reducing gait asymmetry could aid in the recovery of gait function and balance. However, the results of our study did not demonstrate a clear advantage of overground robot exoskeleton gait training over conventional gait training in terms of gait symmetry. Further research is therefore needed to determine the superiority of overground robot exoskeleton gait training in improving gait symmetry in patients with stroke.

### 4.2. Interpretation of PEDro Score and Publication Bias

The participants included in the studies in this analysis were randomly assigned to the experimental and control groups, with an average PEDro score of 6.21, indicating high quality (PEDro scale: 6–10). The PEDro score assesses the internal validity and methodological quality of the literature, and the average score of the studies included in this research was higher than the PEDro database’s average score of 5 [86]. Although blinding of participants and therapists is challenging due to the nature of the intervention, the results of the meta-analysis conducted in this study sufficiently ensure internal validity, with statistical publication bias not detected in the majority of analyses.

Egger’s test has the drawback of reduced power when the number of studies is limited [87]. Therefore, sensitivity tests, such as the trim-and-fill method, were additionally employed and presented in the research results. However, trim-and-fill methods do not elucidate the mechanisms underlying publication bias [88,89]. In fact, these methods analyze data under the assumption of symmetry in the funnel plot, and thus do not explain the factors causing asymmetry in the plot [88]. In this study, publication bias and asymmetry were identified in gait endurance through Egger’s test and funnel plot. Interpretation of the results for this item should be approached with caution when using the trim-and-fill method.

### 4.3. Study Limitations

The primary limitation of this study is the difficulty in generalizing the results due to the inclusion of an insufficient number of studies. Furthermore, despite systematic searches of electronic databases, reporting bias may exist, while subjective judgment in evaluating the data could introduce observer bias. Finally, the use of different types of robotic devices and varying treatment durations across the included studies may lead to different outcomes, potentially biasing the results. Finding solutions to these limitations through further research should provide stronger evidence regarding the effectiveness of overground robot exoskeleton gait training for patients with stroke.

## 5. Conclusions

Overall, this study aimed to investigate the impact of overground robot exoskeleton gait training on the gait outcomes, balance, and motor function of patients with stroke by analyzing the results of existing RCTs through a meta-analysis. The results of the analysis showed that overground robot exoskeleton gait training exhibited some effects on improving gait outcomes in patients with stroke compared to conventional gait training. However, it was not effective at improving balance or motor function. Nevertheless, overground robot exoskeleton gait training offers many advantages, such as safely providing normal gait patterns after stroke onset and reducing gait asymmetry.

The evidence that overground robot exoskeleton gait training can improve gait speed, especially under specific conditions (e.g., sessions exceeding 30 min, conducted three times or less per week, and lasting four weeks or less) suggests that this intervention could be strategically incorporated into stroke rehabilitation programs. Clinicians should consider integrating this technology as part of a comprehensive rehabilitation plan, particularly for patients who demonstrate limited progress with conventional gait training. Moreover, while the intervention may not significantly improve balance or motor function, it can still be valuable for patients who need to focus on gait symmetry and speed. Additionally, the safety profile of overground robot exoskeletons makes them a viable option for early-stage rehabilitation, potentially reducing the risk of injury during gait training.

In summary, provided that appropriate goal-setting and intensity are ensured, overground robot exoskeleton gait training should be considered an effective intervention for improving gait speed and addressing gait asymmetry in patients with stroke. Future studies should explore optimizing training protocols to enhance the balance and motor function benefits of this intervention.

## Figures and Tables

**Table 1:** PEDro scale.

No.	Details of the Contents
1	Eligibility criteria were specified.
2	Subjects were randomly allocated to groups.
3	Allocation was concealed.
4	Groups were similar at baseline regarding the most important prognostic indicators.
5	Blinding of all subjects.
6	Blinding of all therapists who administered the therapy.
7	Blinding of all assessors who measured at least one key outcome.
8	Measures of at least one key outcome were obtained from more than 85% of the subjects initially allocated to groups.
9	All subjects for whom outcome measures were available received the treatment or control condition as allocated or, where this was not the case, data for at least one key outcome was analyzed by “intention to treat”.
10	Results of between-group statistical comparisons are reported for at least one key outcome.
11	Study provides both point measures and measures of variability for at least one key outcome.

**Table 2:** Characteristics of the 19 included randomized controlled trials.

Study /Location	Study Design	Sample Size (n, M/F)	Age (Mean)	Phase of Stroke	Robotic Device	Intervention Group	Control Group	Duration and Frequency of Study Period	Outcomes
Amy et al. [38] /UK	Two-center, 2-arm RCT	Total: 34 Ex ^1^: 16 (14/2) Con ^2^: 18 (14/4)	Ex ^1^: 59.6 ± 10.1 Con ^2^: 65.1 ± 10.1	Chronic stroke	Alter G, Bionic Leg orthosis	Overground robotic-assisted gait-training program	Physical activity	30 min per day, home based (self), 10-weeks	6MWT ^4^, FAC ^5^, BBS ^6^, TUG ^7^
Jayaraman et al. [29] /USA	2-arm RCT	Total: 50 Ex ^1^: 25 Con ^2^: 25	Ex ^1^: 59.5 ± 9.7 Con ^2^: 61.6 ± 12.6	Chronic stroke	Stride Management Assist (SMA)-Honda	Overground gait and functional gait training with the SMA	Functional training based on the participant’s goals	45 min, 3 times a week for 6~8 weeks	10MWT ^3^, 6MWT ^4^, BBS ^6^
Kang et al. [39] /Korea	2-arm RCT	Total: 30 Ex ^1^: 15 (10/5) Con ^2^: 15 (8/7)	Ex ^1^: 64.3 ± 4.6 Con ^2^: 62.9 ± 6.0	Chronic stroke	SUBAR exoskeleton	SUBAR-assisted gait training	Conventional physiotherapy	30 min, 10 sessions for 3 weeks	10MWT ^3^, Gait speed, FAC ^5^, TUG ^7^, BBS ^6^
Lee et al. [40] /Korea	2-arm RCT	Total: 26 Ex ^1^: 14 (7/7) Con ^2^: 12(7/5)	Ex ^1^: 61.85 ± 7.87 Con ^2^: 62.25 ± 6.36	Chronic stroke	Gait Enhancing and Motivating System (GEMS)	Gait training with GEMS	Gait training	45 min, 3 times a week for 4 weeks	Gait speed
Li et al. [41] /China	Multicenter, 2-arm RCT	Total: 130 Ex ^1^: 57 (48/9) Con ^2^: 57 (45/12)	Ex ^1^: 50 Con ^2^: 51.67	Subacute stroke	BEAR-H1 lower limb exoskeleton robot	Locomotor training using the BEAR-H1 exoskeleton robot	Conventional gait training	30 min, two sessions daily, 5 days a week for 4 weeks	6MWT ^4^, FMA-LE ^8^
Louie et al. [42] /Canada	Multicenter, 2-arm RCT	Total: 36 Ex ^1^: 19 (16/3) Con ^2^: 17 (10/7)	Ex ^1^: 59.6 ± 15.8 Con ^2^: 55.3 ± 10.6	Subacute stroke	EksoGT	Exoskeleton intervention	Standard physical therapy	60 min, 3 times a week for until discharge	Gait speed, 6MWT ^4^, FAC ^5^, BBS ^6^, FMA-LE ^8^
Luca et al. [43] /Italy	2-arm RCT	Total: 30 Ex ^1^: 15 (11/4) Con ^2^: 15 (11/4)	Ex ^1^: 54.4 ± 11.9 Con ^2^: 55.8 ± 13.2	Chronic stroke	EksoGT	Robotic gait rehabilitation	Conventional gait training	60 min, 3 times a week for 8 weeks	10MWT ^3^, TUG ^7^
Molteni et al. [44] /Italy	Multicenter, 2-arm RCT	Total: 75 Ex ^1^: 38 (21/17) Con ^2^: 37 (21/19)	Ex ^1^: 62.13 ± 8.75 Con ^2^: 68.24 ± 8.58	Subacute stroke	Ekso™	Gait rehabilitation with Ekso™	Conventional training	60 min, 5 days/week for 3 weeks	10MWT ^3^, 6MWT ^4^, FAC ^5^
Nam et al. [45] /Korea	2-arm RCT	Total: 34 Ex ^1^: 18 (11/7) Con ^2^: 16 (6/10)	Ex ^1^: 48.33 ± 15.56 Con ^2^: 68.56 ± 17.35	Chronic stroke	Exowalk	Electromechanically assisted gait training (Exowalk)	Physical-therapist-assisted gait training	30 min, 5 days a week for 4 weeks	10MWT ^3^, 6MWT ^4^, FAC ^5^, BBS ^6^
Nam et al. [46] /Korea	Multicenter, 2-arm RCT	Total: 38 Ex ^1^: 18 (8/10) Con ^2^: 20 (14/6)	Ex ^1^: 57.3 ± 8.71 Con ^2^: 60 ± 11.48	Chronic stroke	Exowalk	Electromechanically assisted gait training (Exowalk)	Conventional gait training	60 min, 5 times per week for 2 weeks	10MWT ^3^, 6MWT ^4^, FAC ^5^, BBS ^6^
Stein J et al. [47] /USA	2-arm RCT	Total: 24 Ex ^1^: 12 (10/2) Con ^2^: 12 (7/5)	Ex ^1^: 57.6 ± 10.7 Con ^2^: 56.6 ± 15.1	Chronic stroke	Alter G, Bionic Leg orthosis	Group exercise program with robot device	Group exercise program	1 h, 3 times per week for 6 weeks	10MWT ^3^, 6MWT ^4^, BBS ^6^, TUG ^7^
Tanaka et al. [48] /Japan	2-arm RCT	Total: 41 Ex ^1^: 21 (13/8) Con ^2^: 20 (14/6)	Ex ^1^: 64.9 ± 12.2 Con ^2^: 62.3 ± 9.3	Subacute stroke	Stride Management Assist (SMA)-Honda	Robotic-assisted gait training	Conventional gait training	20 min per day for 10 consecutive days	Gait speed
Watanabe et al. [49] /Japan	2-arm RCT	Total: 22 Ex ^1^: 11 (7/4) Con ^2^: 11 (4/7)	Ex ^1^: 67.0 ± 16.8 Con ^2^: 75.6 ± 13.9	Subacute stroke	Robot Suit Hybrid Assistive Limb (HAL)	Gait rehabilitation with the HAL	Conventional gait training	20 min once per day, 3 times per week for 4 weeks	10MWT ^3^, 6MWT ^4^, FAC ^5^, TUG ^7^, FMA-LE ^8^
Watanabe et al. [50] /Japan	2-arm RCT	Total: 24 Ex ^1^: 12 (8/4) Con ^2^: 12 (8/4)	Ex ^1^: 66.9 ± 16.0 Con ^2^: 76.8 ± 13.8	Subacute stroke	Robot Suit Hybrid Assistive Limb (HAL)	Gait training with HAL.	Conventional gait training	20 min once per day, 3 times a week, total of 12 sessions (4 weeks)	10MWT ^3^, 6MWT ^4^, FAC ^5^, TUG ^7^, FMA-LE ^8^
Watanabe et al. [51] /Japan	2-arm RCT	Total: 20 Ex ^1^: 9 (6/3) Con ^2^: 11 (4/7)	Ex ^1^: 60.0 ± 11.7 Con ^2^: 77.4 ± 14.3	Subacute stroke	Robot Suit Hybrid Assistive Limb (HAL)	Gait treatment with HAL.	Conventional gait training	20 min once per day, 3 times per week for 4 weeks	10MWT ^3^, 6MWT ^4^, FAC ^5^, TUG ^7^, FMA-LE ^8^
Xia Li et al. [52] /China	2-arm RCT	Total: 36 Ex ^1^: 17 (15/2) Con ^2^: 15 (14/1)	Ex ^1^: 50.13 ± 9.49 Con ^2^: 50.53 ± 12.26	Subacute stroke	BEAR-H1 lower limb exoskeleton robot	Gait training assisted with the BEAR-H1	Conventional training	30 min, twice a day, 5 days a week for 4 weeks	Gait speed, 6MWT ^4^, FAC ^5^, FMA-LE ^8^
Yeung et al. [28] /Hong Kong	Two-center, 3-arm RCT	Total: 47 Ex ^1^ 1: 14 (8/6) Ex ^1^ 2: 16 (8/8) Con ^2^: 17(8/9)	Ex ^1^ 1: 64.6 ± 12.6 Ex ^1^ 2: 68.3 ± 10.3 Con ^2^: 63.6 ± 5.2	Subacute stroke	Dynamixel MX-106R	Robot-assisted training	Conventional rehabilitation	30 min, 2 session a week for 20 sessions	10MWT ^3^, FAC ^5^, BBS ^6^
Yokota et al. [53] /Japan	2-arm RCT	Total: 37 Ex ^1^: 18 (16/2)Con ^2^: 19 (12/7)	Ex ^1^: 69 Con ^2^: 69	Acute stroke	Robot Suit Hybrid Assistive Limb (HAL)	Gait training with HAL	Gait training	1–3 sessions per day (20 min per session), 3 days a week for 3 weeks	FAC ^5^, FMA-LE ^8^
Yokota et al. [54] /Japan	2-arm RCT	Total: 22 Ex ^1^: 12 (7/5) Con ^2^: 10 (5/5)	Ex ^1^: 65.3 ± 10.1 Con ^2^: 62.5 ± 10.6	Acute stroke	Robot Suit Hybrid Assistive Limb (HAL)	Gait training with HAL	Conventional physical therapy	1–3 sessions per day (20 min per session), 3 days a week for 20 sessions	FAC ^5^, FMA-LE ^8^

^1^ Experimental group. ^2^ Control group. ^3^ 10 m walking test. ^4^ 6 min walking test. ^5^ Functional ambulation category. ^6^ Berg Balance Scale. ^7^ Timed Up and Go Test. ^8^ Fugl–Meyer assessment—lower extremity [28,29,38,39,40,41,42,43,44,45,46,47,48,49,50,51,52,53,54].

**Table 3:** PEDro scale of the included studies (Y—Yes, N—No).

Study (Year)	Concealed Allocation	Baseline Similarity	Subject Blinding	Therapist Blinding	Assessor Blinding	<15% Dropouts	Intention to Treat Analysis	Between-Group Difference Reported	Point Estimate, Variability Reported	Total
Amy et al. [38]	Y	Y	N	N	N	Y (8.82%)	Y	N	Y	7
Jayaraman et al. [29]	N	Y	N	N	N	Y (7%)	N	N	Y	5
Kang et al. [39]	N	Y	N	N	N	Y (7%)	Y	Y	Y	7
Lee et al. [40]	N	Y	N	N	N	Y (0%)	Y	Y	Y	7
Li et al. [41]	N	Y	N	N	N	Y (12.31%)	Y	Y	N	6
Louie et al. [42]	Y	Y	N	N	Y	Y (5%)	Y	Y	Y	9
Luca et al. [43]	Y	Y	N	N	N	Y (0%)	N	N	Y	6
Molteni et al. [44]	N	N	N	N	N	Y (6.25%)	N	N	Y	4
Nam et al. [45]	N	N	N	N	N	N (15%)	N	Y	Y	4
Nam et al. [46]	N	Y	N	N	Y	Y (5%)	N	Y	N	6
Stein J et al. [47]	Y	Y	N	N	Y	Y (0%)	Y	Y	Y	9
Tanaka et al. [48]	N	Y	N	N	N	Y (0%)	Y	Y	Y	7
Watanabe et al. [49]	N	Y	N	N	N	N (31.25%)	N	Y	Y	5
Watanabe et al. [50]	N	Y	N	N	N	N (27.27%)	N	N	Y	4
Watanabe et al. [51]	N	Y	N	N	N	N (39.39%)	N	Y	Y	6
Xia Li et al. [52]	N	Y	N	N	Y	Y (11.11%)	N	Y	Y	7
Yeung et al. [28]	N	Y	N	N	Y	Y (8.51%)	Y	Y	Y	8
Yokota et al. [53]	N	Y	N	N	N	N (21.28%)	N	Y	Y	5
Yokota et al. [54]	N	Y	N	N	N	Y (8.33%)	N	Y	Y	6

**Table 4:** Homogeneity test for gait speed.

	N	*p*-Value	I^2^	Point Estimate	95% CI		Standard Error	Q-Value
					Lower Limit	Upper Limit		
10MWT ^1^	10	0.02	0%	0.28	0.05	0.21	1.72	6.14
Gait analysis	5	0.16	0%	0.22	−0.09	0.53	3.28	4.16
Total	15	0.006	0%	0.26	0.08	0.44	1.51	10.39

^1^ 10 m walking test.

**Table 5:** Subgroup analyses of overground robot exoskeleton gait training on gait speed in 15 trials.

Category	Subgroups	No. of Trials	Sample Size	SMD (d)	95% CI	Heterogeneity *p*-Value of Chi-Square Test (I^2^)	Overall Effect Z Value (*p*-Value)
Region	Asian	11 ^(2, 3, 4, 7, 8, 10, 11, 12, 13, 14)^	319	0.26	0.03, 0.48	5.50 (0%)	2.27 (0.02 *)
	Non-Asian	4 ^(1, 5, 6, 9)^	140	0.28	−0.13, 0.68	4.25 (29%)	1.34 (0.18)
Phase of stroke	Acute	0	0				
	Subacute	7 ^(4, 5, 10, 11, 12, 13, 14)^	197	0.25	−0.04. 0.53	1.24 (0%)	1.70 (0.09)
	Chronic	8 ^(1, 2, 3, 6, 7, 8, 9)^	262	0.28	0.01, 0.55	8.50 (18%)	2.00 (0.05)
Length of training session	≤30 min/session	9 ^(2, 4, 7, 10, 11, 12, 13, 14)^	255	0.18	−0.07, 0.43	2.37 (0%)	1.44 (0.15)
	>30 min/session	6 ^(1, 3, 5, 6, 8, 9)^	204	0.37	0.05, 0.70	6.50 (23%)	2.27 (0.02 *)
Frequency of training	≤3 times/week	9 ^(1, 3, 5, 6, 9, 11, 12, 13, 14)^	254	0.38	0.12, 0.63	6.62 (0%)	2.93 (0.003 *)
	>3 times/week	6 ^(2, 4, 7, 8, 10)^	205	0.12	−0.15, 0.40	1.38 (0%)	0.88 (0.38)
Duration of training	≤4 weeks	10 ^(2, 3, 4, 7, 8, 10, 11, 12, 13)^	288	0.25	0.02, 0.48	5.45 (0%)	2.09 (0.04 *)
	>4 weeks	5 ^(1, 5, 6, 9, 14)^	171	0.28	−0.03, 0.60	4.28 (6%)	1.75 (0.08)

* *p* < 0.05. ^1^ Jayaraman et al., 2019 [29]. ^2^ Kang et al., 2021 [39]. ^3^ Lee et al., 2019 [40]. ^4^ Xie Li et al., 2021 [52]. ^5^ Louie et al., 2021 [42]. ^6^ Luca et al., 2020 [43]. ^7^ Nam et al., 2019 [45]. ^8^ Nam et al., 2020 [46]. ^9^ Stein J et al., 2014 [47]. ^10^ Tanaka et al., 2019 [48]. ^11^ Watanabe et al., 2014 [49]. ^12^ Watanabe et al., 2017 [50]. ^13^ Watanabe et al., 2021 [51]. ^14^ Yeung et al., 2021 [28].

**Table 6:** Homogeneity test for gait endurance.

	N	Studies Trimmed	*p*-Value	I^2^	Point Estimate	95% CI		Standard Error	Q-Value
						Lower Limit	Upper Limit		
Gait endurance	12		0.23	0%	0.11	−0.07	0.28	0.43	3.87
Adjusted value		6			−0.009	−0.15	0.13		10.44

**Table 7:** Homogeneity test for gait ability.

	N	Studies Trimmed	*p*-Value	I^2^	Point Estimate	95% CI		Standard Error	Q-Value
						Lower Limit	Upper Limit		
Gait ability	13		0.07	2%	0.18	−0.01	0.37	1.71	13.03
Adjusted value		1			0.14	−0.05	0.34		15.65

**Table 8:** Homogeneity test for balance.

	N	χ^2^	I^2^	Point Estimate	95% CI		Z	*p*-Value
					Lower Limit	Upper Limit		
Berg Balance Scale	8	0.25	23%	0.05	−0.23	0.32	0.34	0.74
Timed Up and Go Test	7	0.43	0%	−0.15	−0.42	0.15	0.96	0.34

**Table 9:** Homogeneity test for motor function.

	N	χ^2^	I^2^	Point Estimate	95% CI		Z	*p*-Value
					Lower Limit	Upper Limit		
Fugl–Meyer Assessment—Lower Extremity	9	0.20	28%	0.21	−0.05	0.47	1.56	0.12

## Data Availability

The data presented in this study are available upon reasonable request from the corresponding author.
